# Dirichlet process model for joint haplotype inference and GWAS

**DOI:** 10.1186/1753-6561-6-S6-P49

**Published:** 2012-10-01

**Authors:** Avinash Das Sahu, Sridhar Hannenhalli

**Affiliations:** 1 University of Maryland, College Park, MD 20740, USA; 2 University of Maryland Institute for Advanced Computer Studies, MD, USA

## 

Identification of causal genomic mutations that underlie disease phenotypes remains a key problem in the field of medical informatics. With the advent of new sequencing technologies and decreasing cost of human genotyping, it is now possible to study genotype-phenotype interactions, such as genome-wide association studies (GWAS), at the population level. However, due to large genomic variance and linkage disequilibrium, genetic diversity of a complete human population cannot be captured by a limited number of clusters. Furthermore, application of current haplotype inferencing (phasing) methods to rare genomic variance, such as disease-related alleles, is not reliable. Hence, a satisfactory method for deleterious mutation identification remains largely elusive. Here we present a non-parametric Bayesian model that jointly infers haplotypes and identifies deleterious mutations, taking into consideration genomic variance in the human population. The model is based on the Dirichlet process, which can capture genomic variance by modeling it with non-bounded numbers of clusters.

